# Dabrafenib and trametinib vs anti-PD(L)1 for the adjuvant treatment of locally advanced BRAF-mutant melanoma: a systematic review and meta-analysis

**DOI:** 10.1093/oncolo/oyaf247

**Published:** 2025-08-04

**Authors:** Daniel V Araujo, Bruno Lins Souza, Mariana F Seibel, Aline F Fares, Vitor T Liutti

**Affiliations:** Division of Hematology and Medical Oncology, Department of Medicine, University of Florida, Gainesville, FL 32610, United States; Department of Medicine, Federal University of Ceará, Fortaleza, CE 60430-160, Brazil; Division of Medical Oncology, Federal University of Health Sciences of Porto Alegre (UFCSPA), Porto Alegre, RS 90050-170, Brazil; Division of Hematology and Medical Oncology, Department of Medicine, University of Florida, Gainesville, FL 32610, United States; Division of Medical Oncology, Hospital de Cancer de Londrina, Londrina, PR 86015-520, Brazil

**Keywords:** melanoma, stage III, BRAF mutant, dabrafenib and trametinib, anti-PD1, nivolumab, pembrolizumab

## Abstract

**Background:**

Both dabrafenib and trametinib (D + T) and anti-PD(L)1s have been shown to improve recurrence-free survival (RFS) in patients with stage III or resected stage IV BRAF-mutant melanoma. However, no randomized controlled trials (RCTs) have directly compared them in the adjuvant setting, creating uncertainties about the optimal approach. This systematic review and meta-analysis address this knowledge gap.

**Methods:**

A comprehensive search of PubMed, Embase, and Scopus was conducted to identify studies comparing D + T with anti-PD(L)1 therapies. Studies with overlapping populations were excluded. Statistical analyses employed a random-effects model, with heterogeneity assessed via *I* ^2^ statistics. This study was registered with PROSPERO (CRD42024553421).

**Results:**

Eight observational studies (2394 patients) met the inclusion criteria. No eligible RCTs were identified. Median follow-up ranged from 10 to 53 months. Dabrafenib and trametinib improved RFS compared to anti-PD(L)1 therapies (hazard ratio [HR] 0.53, 95% CI, 0.40-0.70, *P* < .01; *I* ^2^ = 55%). However, no significant difference was observed in overall survival (OS) (HR 0.83, 95% CI, 0.60-1.15, *P* = .27; *I* ^2^ = 0%). Subgroup and sensitivity analyses yielded similar results. Dabrafenib and trametinib was associated with a higher rate of treatment discontinuation due to adverse events (AEs), with a relative risk of 1.57 (95% CI, 1.30-1.91, *P* < .01; *I* ^2^ = 0%), corresponding to a risk difference of 8% (95% CI, 5%-12%, *P* < .01; *I* ^2^ = 0%).

**Conclusions:**

Dabrafenib and trametinib demonstrated superiority over anti-PD(L)1 therapies in terms of RFS. However, no OS benefit was observed, and D + T was associated with a higher risk of treatment discontinuation. These findings should be considered when counseling patients, as the choice of adjuvant therapy may need to be tailored to individual preferences and tolerability.

Implications for practice Dabrafenib and trametinib is superior to anti-PD(L)1 therapies for the adjuvant treatment of BRAF mutant melanoma in terms of RFS. However, no OS difference has been demonstrated, and D + T is associated with a higher risk of treatment discontinuation due to AEs. These findings highlight the need for individualized treatment decisions based on patient preferences and tolerability.

## Introduction

Melanoma is among the most aggressive forms of skin cancer, with significant morbidity and mortality. Approximately 50% of melanomas harbor a BRAF mutation, with the most common being V600E, followed by V600K and other less frequent variants.^1^ In the metastatic setting, targeted therapy with BRAF/MEK inhibitors (eg, dabrafenib plus trametinib, vemurafenib plus cobimetinib, or encorafenib plus binimetinib) is approved for patients whose tumors carry a V600E or V600K mutation.^2–4^ Emerging data suggest that BRAF/MEK inhibitors may also be effective against certain non V600E/K BRAF mutations, particularly those classified as Tier 1 in actionability.^5–7^

Another cornerstone of treatment for metastatic melanoma is immunotherapy with anti-PD-1-based immune checkpoint inhibitors (ICIs). These agents are “genomically agnostic,” meaning their efficacy is not contingent on a specific mutation profile, and they can be administered as monotherapy or in combination with anti-CTLA-4 or anti-LAG-3 agents.^8–10^ Notably, the combination of nivolumab and ipilimumab has demonstrated numerically superior progression-free survival (PFS) and overall survival (OS) compared to nivolumab alone (although this was an exploratory comparison), with particular benefit in patients with BRAF-mutant melanoma.^8^ Furthermore, nivolumab plus ipilimumab was shown to be superior to dabrafenib plus trametinib as first-line therapy in patients with BRAF V600E/K-mutant metastatic melanoma,^11^^,^^12^ suggesting that combination immunotherapy may be a preferred option whenever feasible. Likewise, the addition of an anti-LAG-3 agent (relatlimab) to nivolumab improved PFS compared to nivolumab monotherapy in metastatic melanoma, with a trend toward improved OS.^10^ Despite these promising findings, the question of how anti-PD-1 monotherapy alone compares to BRAF/MEK inhibition in BRAF-mutant metastatic melanoma remains incompletely resolved.

In the adjuvant setting, where treatment is given after surgical resection of high-risk melanoma, the therapeutic goal shifts to reducing recurrence risk and improving survival. Stage III melanoma is defined by regional lymph node involvement or in-transit metastases/satellite lesions and carries a substantial risk of relapse. For patients with stage III melanoma harboring a BRAF mutation, one year of adjuvant therapy with dabrafenib plus trametinib or an anti-PD-1 agent (nivolumab or pembrolizumab) significantly prolongs recurrence-free survival (RFS).^13–16^ Nivolumab is also approved for patients with resected stage IV disease.^16^ However, unlike in the metastatic setting, the combination of nivolumab and ipilimumab failed to show superiority over nivolumab alone in the adjuvant setting and is therefore not recommended for most patients.^17^ Despite the clear RFS advantages of adjuvant therapy—whether targeted therapy or immunotherapy—no significant improvement in OS has been demonstrated to date in pivotal trials.^13–16^ Real-world data have also yielded conflicting findings regarding the OS benefit of adjuvant treatment, as evidenced by reports from Sweden and the United States.^18^^,^^19^ Interpreting OS in the adjuvant context is particularly challenging because subsequent treatments at the time of relapse can dramatically influence survival outcomes. Notably, approximately half of patients receiving first-line nivolumab plus ipilimumab in the metastatic setting remain alive after 10 years,^8^ underscoring how later lines of therapy may confound OS analyses in the adjuvant setting.

To date, no randomized trials have directly compared adjuvant BRAF/MEK inhibition with anti-PD-1 therapy in patients with BRAF-mutant stage III or resected stage IV melanoma. In most countries, both strategies are approved and reimbursed for this population, leaving clinicians and patients to weigh the benefits, risks, and side-effect profiles of each approach in the absence of head-to-head data. Therefore, we conducted a systematic review and meta-analysis to compare the efficacy of these two adjuvant treatment modalities in BRAF-mutant stage III and resected stage IV melanoma.

## Methods

### Design and setting

This systematic review and meta-analysis were conducted and reported in accordance with the Cochrane Collaboration Handbook for Systematic Review of interventions and the Preferred Reporting Items for Systematic Reviews and Meta-Analysis guidelines.^20^ The study protocol was prospectively registered in the PROSPERO database (CRD42024553421). Both randomized controlled trials and observational studies were permitted.

### Search strategy

A comprehensive search strategy was performed in PubMed (MEDLINE), EMBASE, and Scopus from inception to June 2024. The search strategy was tailored to each database, but in general involved the following terms and Boolean operators: Melanoma OR “Melanoma”[Mesh]) AND (BRAF OR “BRAF mutant”)) AND (“Stage III” OR “locally advanced” OR resected OR adjuvant)) AND (Dabrafenib OR Trametinib OR Pembrolizumab OR Nivolumab OR BRAF/MEKi OR “BRAF/MEK inhibitor” OR ICI OR Anti-PD1 OR AntiPD1 OR “Immune Checkpoint Inhibitors”[Mesh].

### Eligibility criteria

Inclusion in this meta-analysis was restricted to studies that met all the following eligibility criteria: (1) randomized controlled trials or nonrandomized studies; (2) direct comparison between dabrafenib + trametinib vs anti-PD1; (3) patients with BRAF V600E/K mutant stage III or resected IV melanoma receiving adjuvant treatment; and (4) presenting extractable data on RFS, OS or treatment discontinuation due to adverse events (AEs). Studies were excluded if they fell under the following categories: (1) limited to abstracts without full-text availability and (2) studies with overlapping populations.

### Data extraction

Data extraction was performed independently by 2 reviewers (D.V.A. and V.T.L.) using a predefined protocol. Discrepancies were resolved by consensus. When hazard ratios (HRs) were not directly reported, they were estimated from survival curves using the method described by Parmar et al.^21^ Otherwise, HRs were extracted directly from published records. We applied a hierarchical approach to HR selection, prioritizing those from adjusted analyses (eg, propensity score or multivariable models), followed by univariable HRs, and lastly, HRs derived from survival plots. The data presented in Table 1 reflect the participants who contributed to the effect estimates. Therefore, for studies that employed propensity score matching, the table reports data from the matched cohort rather than the overall study population.

**Table 1.** Characteristics of selected studies.

Study	Population	Design	Comparison	Number of pts	Follow up	Female	Age	BRAF	Stage
Bai et al.^23^	Resected stage III BRAF V600mut melanoma. Neoadjuvant treatment was exclusion AJCC 8^th^ ed. Used for staging.	Retrospective cohort	Dabrafenib + trametinib × Anti PD-1 (pembro/nivo/toripalimab)	393 (65%) D + T 205 Anti PD-1 (34%)	D + T: 29 m (IQR 21-43) Anti PD-1: 38 (IQR: 29-50)	186 (47%) vs 86 (42%)	D + T: 56 (IQR 21) Anti-PD1: 55 (IQR 24)	V600E—501 (83%): 323 (82%) vs 178 (87%) V600K—57 (9.5%): 35 (9%) vs 22 (11%) Others—8 (1.3%): 5 (1%) vs 3 (1%) Unknown—32 (5.3%): 30 (8%) vs 2 (1%)	IIIA—86 (14%): 60 (15%) vs 26 (13%) IIIB—182 (30%): 120 (30%) vs 62 (30%) IIIC—295 (49%): 189 (48%) vs 106 (52%) IIID—29 (4,8%): 20 (5%) vs 9 (4%) III NOS—6 (1%): 4 (1%) vs 2 (1%)
De Meza et al.^24^	Stage III BRAFmut patients. Propensity score matched (optimal matching) AJCC 8^th^ ed. Used for staging.	Retrospective cohort	Dabrafenib + trametinib × anti PD-1	114 (50%) D + T 114 Anti PD-1 (50%)	D + T: 10.8 m (IQR 4.6-17.8) Anti PD-1 13.9 (IQR: 7.3-20.5)	100 (48.3%): 51 (44.7%) vs 39 (43%)	D + T: <65: 76 (66.7%) Anti-PD1: <65: 71 (62.3%)	^a^ V600E—167 (74.5%): 87 (77.7%) vs 80 (71.4%) V600K—24 (10.7%): 11 (9.8%) vs 13 (11.6%) Others - 23 (10.3%): 9 (8%) vs 14 (12.5%) Unknown—10 (4.5%): 5 (4.5%) vs 5 (4.5%)	IIIA—37 (16.2%): 18 (15.8%) vs 19 (16.7%) IIIB—62 (27.2%): 31 (27.2%) vs 31 (27.2%) IIIC/D—101 (44.3%): 51 (44.7%) vs 50 (43.9%) III NOS—28 (12.3%): 14 (12.3%) vs 14 (12.3%)
Haist et al.^25^	Stage III. BRAF mut. Pts w/ongoing treatment and FU < 11, and off treatment for other reasons then intolerance or tumor recurrence and FU < 6 m were excluded. AJCC 8^th^ ed. Used for staging.	Retrospective cohort	Dabrafenib + trametinib × Anti PD-1 (pembro/nivo)	273 (53%) D + T 242 Anti PD-1 (47%)	20 m (95%CI, 18.5-21.5)	^b^228 (46.1%): 108 (45.8%) vs 120 (46.3%)	^b^D + T: 59 (95%CI, 55.2-58.7) Anti-PD1: 56 (95%CI, 55.2-58.7)	^b^V600E: 373 (75.3%): 189 (80.1%) vs 184 (71%) V600K: 56 (11.3%): 29 (12.3%) vs 27 (10.4%) Others: 13 (2.6%): 1.3% vs 10 (3.9%) NOS: 53 (10.7%): 15 (6.4%) vs 38 (14.7%)	^b^IIIA—75 (15.2%): 37 (15.7%) vs 38 (14.7%) IIIB—189 (38.2%): 83 (35.2%) vs 106 (40.9%) IIIC—207 (41.8%): 106 (44.9%) vs 101 (39%) IIID—19 (3.8%): 10 (4.2%) vs 9 (3.5%) NOS: 0 vs 5 (1.9%)
Lodde et al.^26^	Stage III and IV resected. Regardless of BRAF status (only BRAFmut included here). AJCC 8^th^ ed. Used for staging.	Retrospective cohort	Dabrafenib + trametinib × anti PD-1	110 (47%) D + T 122 Anti PD-1 (52%)	D + T: 25.4 m (IQR 20.4-28.8) Anti PD-1 25.9 m (IQR 21.8-31.4)	99 (42%): 50 (45.5%) vs 49 (40.2%)	D + T: 52.1 (IQR 25.7) Anti-PD1: 55.4 (IQR 19.3)	V600E—197(85%): 96 (87.3%) vs 101 (82.9%) V600K—21 (9%): 9 (8.2%) vs 12 (9.8%) Others—12 (5%): 5 (4.5%) vs 7 (5.7%) Unknown—2(1%): 0 vs 2 (1.6%)	IIIA—16 (7%): 7 (6.4%) vs 9 (7.4%) IIIB—88 (38%): 37 (33.6%) vs 51 (41.8%) IIIC—119 (51%): 61 (55.5%) vs 58 (47.5%) IIID– 9 (4%): 5 (4.5%) vs 4 (3.3%)
Placzke et al.^27^	Stage III and IV resected. Regardless of BRAF status (only BRAFmut included in the table) AJCC 8^th^ ed. Used for staging.	Retrospective cohort	Dabrafenib + trametinib × anti PD-1 (pembro/nivo)	101 (65%) D + T 54 Anti PD-1 (34%)	13.9 m (SD: 6.5)	NA	NA	NA	NA
Rigo et al.^28^	Stage III and IV resected. Regardless of BRAF status (only BRAFmut included in the table) AJCC 8^th^ ed. Used for staging.	Retrospective cohort	Dabrafenib + trametinib × anti PD-1 (pembro/nivo)	20 (27%) D + T 54 Anti PD-1 (73%)	NA	NA	NA	V600E/K 74 (100%)	NA
Schumann et al.^29^	Stage III and IV resected. Regardless of BRAF status (only BRAFmut included in the table). AJCC 8^th^ ed. Used for staging.	Retrospective cohort	Dabrafenib + trametinib × anti-PD-1 (nivolumab/pembrolizumab)	D + T 195 (36%) Anti-PD-1 347 (64%)	D + T: 11 m (0-41 m) Nivo: 14 m (0-45 m) Pembro: 14 m (0-53 m)	NA	NA	NA	NA
Zhong et al.^30^	Stage III. BRAF V600mut. AJCC 8^th^ ed. Used for staging.	Retrospective cohort	Dabrafenib + trametinib × anti-PD-1 (toripalimab/pembrolizumab)	D + T 25 (50%) Anti-PD-1 25 (50%)	D + T: 11 m Anti-PD1: 22 m	31 (62%): 15 (60%) vs 16 (64%)	D + T–<60 y: 17 (68%) Anti-PD-1–<60 y: 18 (72%)	100% V600E	IIIA: 6 (12%): 3 (12%) vs 3 (12%) IIIB: 5 (10%): 3 (12%) vs 2 (8%) IIIC: 30 (60%): 14 (56%) vs 16 (64%) IIID: 5 (10%): 3 (12%) vs 2 (8%) NOS: 4 (8%): 2 (8%) vs 2 (8%)

a Used nearest neighbor caliper matching (as opposed to optimal matching). All the remainder data were from optimal matching.

b Baseline patient characteristics of the cohort who received adjuvant therapy outside of clinical trials and not for resected IV disease (total of patients = 495).

Abbreviations: D + T, dabrafenib and trametinib; IQR, interquartile range; Mut, mutated; Nivo, Nivolumab; .NOS, not otherwise specified; Pembro, pembrolizumab.

### Primary outcomes

Outcomes included RFS, OS, and percentage of treatment discontinuation due to AEs.

### Subgroup and sensitivity analyses

A subgroup analysis including patients with stage IIIA disease was performed. In addition, a sensitivity analysis was performed, restricting the inclusion to only studies with HRs extractable from the manuscript (excluding studies with HRs estimated from survival curves).

### Quality assessment

Non-randomized studies were assessed through the Risk Of Bias In Non-randomized Studies of Interventions tool,^22^ which contains 7 domains and categorizes studies as having low, moderate, serious, critical, or unclear risk of bias. There were no RCTs included in this meta-analysis.

### Statistical analysis

Using the random-effects model, HR, risk ratios (RRs), and risk difference (RD) with 95% CIs were used to compare treatment effects. Heterogeneity was assessed using *I*^2^ statistics, with >25% considered indicative of significant heterogeneity. Review Manager 5.4.1 (Cochrane Center, The Cochrane Collaboration) was used to perform the statistical analysis.

## Results

**Figure 1.**
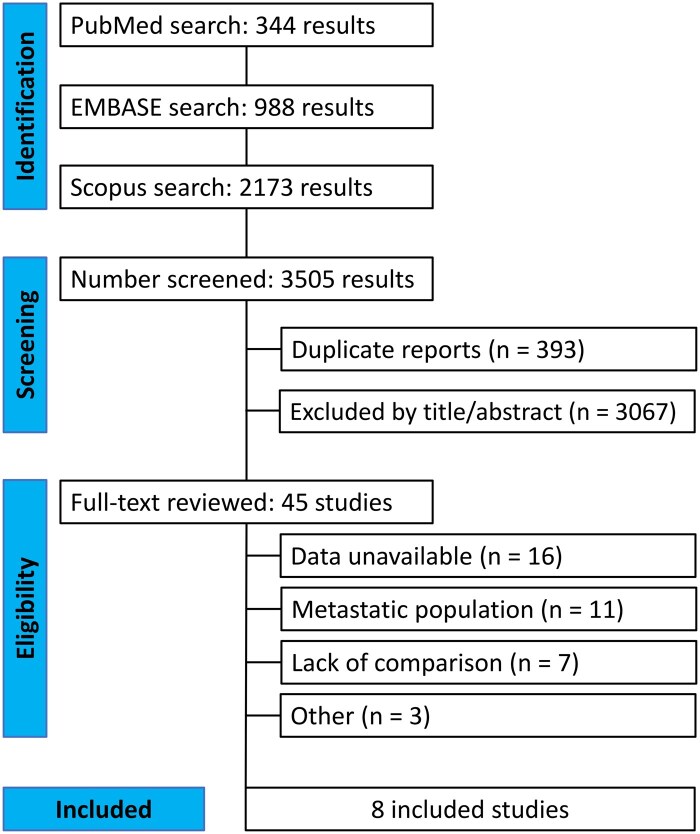
PRISMA flow diagram of study screening and selection. PRISMA, Preferred Reporting Items for Systematic Reviews and Meta-Analysis.

We identified 3505 studies, of which 393 were duplicates, leaving 3112 unique studies. After screening titles and abstracts, we excluded 3067 studies, resulting in 45 studies for full-text review. Of those, 16 were excluded due to unavailability of data, 11 due to focusing on a metastatic population, 7 due to lack of direct comparison between D + T vs anti-PD1, 1 was a review article, and 2 had overlapping populations. Ultimately, 8 studies were included,^23–30^ comprising a total of 2394 patients (Figure 1). Table 1 summarizes the characteristics of the selected studies. Among these, 1231 patients received D + T, while 1163 were treated with anti-PD1 therapy. Most studies compared D + T with either nivolumab or pembrolizumab, while 2 studies also allowed toripalimab as a comparator. All studies were retrospective. The majority included only patients with stage III disease, and in most studies, BRAF V600E was the most commonly reported BRAF mutation.

### Recurrence-free survival

**Figure 2.**
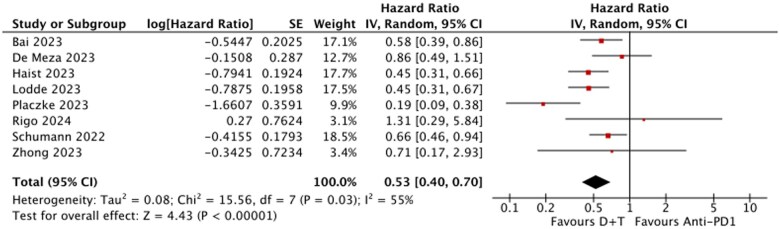
Recurrence-free survival (RFS) D + T vs anti-PD(L)1s. D + T, dabrafenib and trametinib.

Adjuvant D + T was associated with a significant improvement in RFS compared to anti-PD1 across the overall population. The pooled HR for RFS favored D + T, with an HR = 0.53 (95% CI, 0.40-0.70; *P* < .00001), indicating a 47% relative reduction in the hazard of recurrence. Moderate heterogeneity was observed across studies (*I*^2^ = 55%). Figure 2 illustrates the findings.

To further explore the consistency of this effect in lower-risk disease, we performed a subgroup analysis with only patients with stage IIIA melanoma. In this subgroup, D + T continued to demonstrate a RFS benefit in comparison to anti-PD1: HR = 0.42 (95% CI, 0.28-0.63; *P* < .0001), translating to a 58% relative reduction in hazard risk. Notably, no heterogeneity was detected in this subgroup analysis (*I*^2^ = 0%) (Figure S1).

### Overall survival

**Figure 3.**
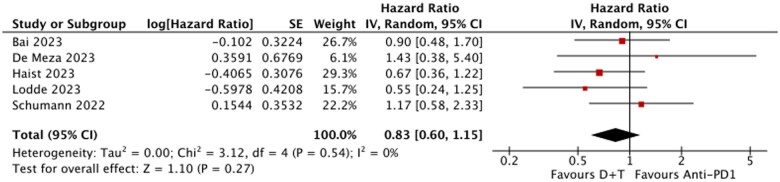
Overall survival (OS) D + T vs anti-PD(L)1s.  + T, dabrafenib and trametinib.

No significant differences in OS were observed between patients treated with D + T and anti-PD1. The pooled HR for OS was HR = 0.83 (95% CI, 0.60-1.15; *P* = .27). We found no heterogeneity among the included studies (*I*^2^ = 0%), suggesting consistent findings across trials. Figure 3.

### Sensitivity analyses

To confirm our findings, we performed a sensitivity analysis including only studies with HRs directly abstracted from the manuscripts. We found consistent results. For RFS, we found HR = 0.56 (95% CI, 0.46-0.70; *P* < .00001), *I*^2^ = 27%, favoring D + T over anti-PD1 (Figure S2). For OS, we found HR = 0.75 (95% CI, 0.46-1.24; *P* = .26), *I*^2^ = 0%, favoring D + T over anti-PD1 (Figure S3).

### Discontinuation due to AEs

**Figure 4.**
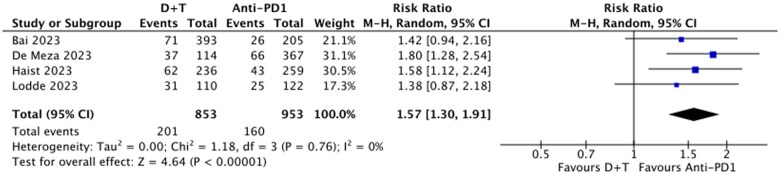
Relative ratio (RR) of discontinuing treatment due to adverse events, D + T vs anti-PD(L)1s. D + T, dabrafenib and trametinib.

**Figure 5.**
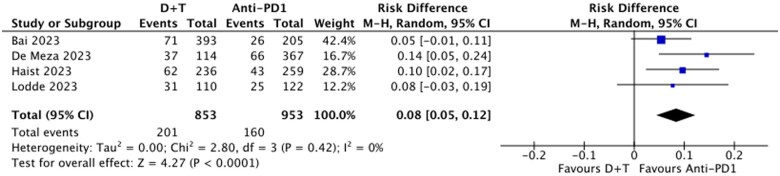
Risk difference of discontinuing treatment due to adverse events, D + T vs anti-PD(L)1s. Ds + T, dabrafenib and trametinib.

Dabrafenib and trametinib was associated with a higher rate of treatment discontinuation due to AEs, with discontinuation rates of 24% for D + T and 16% for anti-PD1. The relative risk (RR) of discontinuation due to AEs was 1.57 (95% CI, 1.30-1.91, *P* < .01; *I*^2^ = 0%) (Figure 4), corresponding to a RD of 8% (95% CI, 5%-12%, *P* < .01; *I*^2^ = 0%) (Figure 5).

### Quality assessment

**Figure 6.**
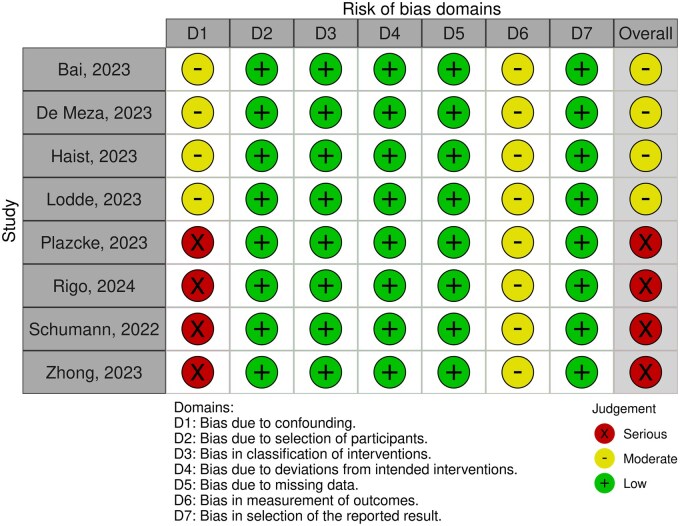
Risk of bias.

Individual appraisal of each study included in this meta-analysis for the primary endpoint (RFS) is outlined in Figure 6. All the 8 included studies were non-randomized. Four studies were considered as having moderate and 4 as having high risk of bias in the confounding domain, and all studies were considered as having moderate risk of bias in the measurement of outcomes domain.

## Discussion

In this systematic review and metanalysis of 8 studies, we found that adjuvant D + T is associated with a favorable RFS in comparison to anti-PD1s in patients with locally advanced BRAF mutant melanoma. However, this benefit did not translate into an OS advantage, albeit a non-statistical trend in favor of D + T. Subgroup and sensitivity analyses reinforced these findings, adding robustness to our conclusions. Interestingly, patients receiving D + T had a higher risk of treatment discontinuation due to AEs compared to those treated with anti-PD1 therapy.

Both D + T and anti-PD1s are standard of care options for the adjuvant treatment of stage III and resected stage IV BRAF mutant melanoma.^13–16^ Both treatments have been shown to prolong RFS in comparison to placebo.^13–15^ However, both D + T and anti-PD1s failed to demonstrate OS improvements.^13–16^ It has been speculated that advancements in systemic therapies for metastatic melanoma, including ICIs and targeted therapies, allow a significant proportion of relapsed patients to be successfully rescued.^8^ Nonetheless, reducing recurrence rates remains clinically meaningful, as it can positively impact patients’ quality of life. Furthermore, systemic therapies in the metastatic setting frequently involve a combination of 2 ICIs, which are associated with a higher risk of developing life-long toxicities.^31^

Dabrafenib and trametinib was superior to anti-PD1s in terms of RFS. A subgroup analysis of stage IIIA patients arrived at similar conclusions. Additional subgroup analyses based on BRAF mutation type (V600E, V600K, or others) and disease stage (stage III vs resected stage IV) were planned but could not be conducted due to insufficient data. The study by Bai et al.^23^ which included the largest reported cohort on this topic, demonstrated that the RFS benefit of D + T over anti-PD1 therapy was consistent across all tested subgroups.

Regarding treatment discontinuation due to AEs, patients on D + T were more likely to stop treatment compared to those receiving anti-PD1 therapy. However, it is crucial to consider the differences in toxicity profiles. Dabrafenib and trametinib is associated with a high incidence of side effects,^32^ with over 60% of patients experiencing fever, often leading to treatment interruptions. Fortunately, symptoms typically resolve quickly upon discontinuation. In contrast, most patients tolerate anti-PD1 therapy quite well with minimal toxicities, though 10%-20% develop grade 3-4 immune-related toxicities.^14–16^ While these are generally manageable with immunosuppressive therapy, severe complications such as immune-related myocarditis^33^ or insulin-dependent diabetes,^34^ despite rare, can have lifelong consequences. Furthermore, a non-negligible percentage of patients experiencing immune-related AEs may experience chronic immune-related AEs.^35^^,^^36^ These risks should be carefully weighed during patient counseling, particularly since some individuals may be cured with surgery alone and might not require adjuvant therapy.

Recently, both D + T and anti-PD1 therapy have been investigated in the neoadjuvant setting, yielding promising results.^37^ Emerging data suggest that ICIs may be more effective when administered in the presence of a minimal tumor burden, as this environment fosters a more robust immune response. This hypothesis is supported by translational studies demonstrating increased effector T-cell infiltration in neoadjuvant-treated tumors compared to adjuvant-treated ones,^38^ as well as by clinical trials.^39^^,^^40^ For instance, the SWOG S1801 trial compared neoadjuvant vs adjuvant pembrolizumab and reported significantly improved event-free survival (EFS) in the neoadjuvant arm (2-year EFS: 72% vs 49%, *P* = .004).^39^ Similarly, the NADINA trial evaluated neoadjuvant nivolumab plus ipilimumab vs adjuvant nivolumab and observed a 12-month EFS of 83.7% vs 57.2%, *P* < .001.^40^ Notably, the CheckMate-915 trial,^17^ which compared the same ICI combination to nivolumab alone in the adjuvant setting, failed to show any RFS benefit, further supporting the notion that ICI efficacy is enhanced in the neoadjuvant context. Neoadjuvant D + T has also been explored, with studies reporting pathologic complete response (pCR) rates of approximately 50%.^41^ However, observational data suggest that RFS and OS outcomes for D + T-treated patients are inferior to those of anti-PD1 therapy.^37^ Consequently, neoadjuvant D + T is becoming a less favored strategy.

While neoadjuvant ICIs appear promising, particularly for patients with a high disease burden, they are not without risks—most notably, the potential for long-term immune-­related toxicities. For patients with lower tumor burden (eg, stage IIIA), who already have a high likelihood of cure with surgery alone,^42^ the risks of neoadjuvant ICIs may outweigh the benefits. In such cases, a more predictable adjuvant treatment approach, such as D + T, may be a safer option.

Our study has several limitations. First, all included studies were non-randomized, carrying inherent biases. However, given the current clinical equipoise between D + T and anti-PD1 therapy, we do not believe treatment selection was strongly influenced by patient characteristics. Second, 4 studies did not report HRs for the primary outcomes, requiring HRs to be extracted from Kaplan-Meier curves. To address this, we conducted a sensitivity analysis including only studies that reported HRs directly in the manuscript, which yielded consistent results, reinforcing our conclusions. Third, there was significant heterogeneity in RFS outcomes, which we partially addressed through subgroup analyses. Unfortunately, due to data limitations, further stratified analyses were not possible.

## Conclusions

Dabrafenib and trametinib demonstrated superiority over anti-PD(L)1 therapies in terms of RFS. However, no OS benefit was observed, and D + T was associated with a higher risk of treatment discontinuation. These findings should be considered when counseling patients, as the choice of adjuvant therapy may need to be tailored to individual preferences and tolerability.

## Supplementary Material

oyaf247_Supplementary_Data

## Data Availability

Data are available upon reasonable request.
