# Supplementary material for: Dabrafenib and trametinib vs anti-PD(L)1 for the adjuvant treatment of locally advanced BRAF-mutant melanoma: a systematic review and meta-analysis
Source: Oncologist. 2025 Aug 4;30(9):oyaf247. doi: 10.1093/oncolo/oyaf247 (PMC12449075; doi:10.1093/oncolo/oyaf247)
Supplement: oyaf247_Supplementary_Data [file oyaf247_supplementary_data.zip › Supplementary Appendix 09Jun2025.docx]

Figure S1: Recurrence free survival (RFS) subgroup analysis of patients with stage IIIA.

Figure S2: Sensitivity analysis of recurrence free survival (RFS) including only studies with data extracted from manuscripts.

Figure S3: Sensitivity analysis of overall survival (OS) including only studies with data extracted from manuscripts.
