# Supplementary figures and images for: Dabrafenib and trametinib vs anti-PD(L)1 for the adjuvant treatment of locally advanced BRAF-mutant melanoma: a systematic review and meta-analysis
Source: Oncologist. 2025 Aug 4;30(9):oyaf247. doi: 10.1093/oncolo/oyaf247 (PMC12449075; doi:10.1093/oncolo/oyaf247)

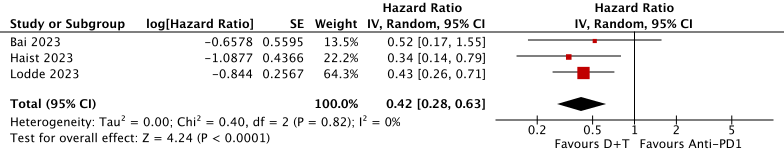

Supplement: oyaf247_Supplementary_Data [file oyaf247_supplementary_data.zip › Fig S1 - RFS IIIA.png]

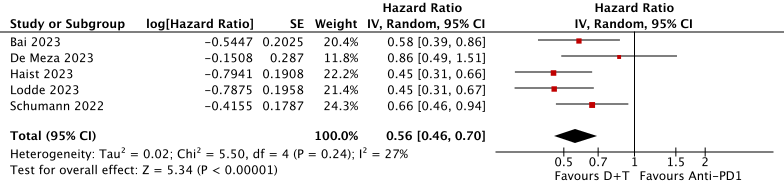

Supplement: oyaf247_Supplementary_Data [file oyaf247_supplementary_data.zip › Fig S2 - RFS non extracted.png]

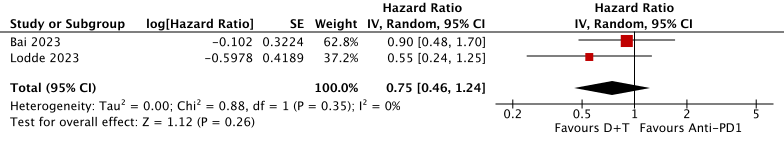

Supplement: oyaf247_Supplementary_Data [file oyaf247_supplementary_data.zip › Fig S3 - OS non extracted.png]
